# A Nonfunctioning Pituitary Macroadenoma Presenting as Cyclical Vomiting and Severe Hyponatremia in a Young Female

**DOI:** 10.1155/2021/5570539

**Published:** 2021-03-31

**Authors:** Dinuka S. Warapitiya, Dimuthu Muthukuda, W. A. H. P. Sanjeewa, Kushalee Poornima Jayawickreme, Shyama Subasinghe

**Affiliations:** ^1^Postgraduate Institute of Medicine, University of Colombo, Colombo, Sri Lanka; ^2^Diabetes and Endocrinology Unit, Sri Jayewardenepura General Hospital, Nugegoda, Colombo, Sri Lanka; ^3^Sri Jayewardenepura General Hospital, Nugegoda, Colombo, Sri Lanka

## Abstract

**Introduction:**

Recurrent vomiting is a commonly overlooked debilitating symptom which causes significant impact on the quality of life. There are several causes for vomiting, ranging from commonly known causes to rare causes. Nonfunctioning pituitary macroadenomas generally present with visual disturbances, headache, and symptoms due to anterior pituitary hormone deficiencies. This case report is about an atypical presentation of a nonfunctioning pituitary macroadenoma in which the patient presented with cyclical vomiting with severe hyponatremia. *Case Report*. A 23-year-old girl presented with four to five episodes of vomiting per day for two days duration. She had a history of similar episodes of vomiting since 2016, with each episode generally lasting for 4-5 days and occurring in every four to six months. All episodes exhibited similar symptomatology and she was free of symptoms in-between. Generalized body weakness, postural dizziness, reduced appetite, and secondary amenorrhea were other symptoms she has had since 2016. Examination findings showed a low body mass index (BMI) (16 kg/m^2^) with normal system examination. Investigations showed severe hyponatremia (110 mmol/L) with hypokalemia (3.2 mmol/L) and hypochloremia (74 mmol/L). Her urinary excretion of potassium, sodium, and serum osmolality was low. Urine osmolality was mildly elevated compared to serum osmolality. Blood urea was normal. Severe hyponatremia with minimal hyponatremic symptoms was suggestive of chronic hyponatremia, which was accentuated by ongoing vomiting and possible reduced intake of salt. Further investigations showed evidence of secondary hypoadrenalism, central hypothyroidism, hypogonadotropic hypogonadism, and mild hyperprolactinemia. Magnetic resonance imaging (MRI) revealed a pituitary macroadenoma with mass effect on the optic chiasma. Hydrocortisone and levothyroxine were started, and she underwent transsphenoidal resection of the pituitary tumor. She recovered from cyclical vomiting.

**Conclusion:**

There can be multiple overlapping aetiologies for every observed symptom, sign, and abnormal investigation finding. Therefore, aetiological diagnosis is challenging, especially in the presence of an atypical clinical presentation. Cyclical vomiting and severe hyponatremia are atypical presentations of nonfunctioning pituitary macroadenomas.

## 1. Introduction

Clinically nonfunctioning pituitary adenoma is a common type of tumor in the sellar region [1]. The prevalence of nonfunctioning pituitary adenomas varies between 60 and 100 cases per million population [1, 2]. It has a bimodal peak incidence between the ages of 25–45 years and 60–70 years and the incidence rate is 1.02–1.08 per 100000 [1, 2]. There is an equal incidence among both genders [1, 2].

These lesions are usually found incidentally (mainly microadenoma) or diagnosed based on symptoms and signs of anterior pituitary hormone deficiency and compressive symptoms such as headache and visual field defects [1].

We are presenting an atypical presentation of nonfunctioning pituitary macroadenomas.

This case is of a Sri Lankan law student with various psychosocial problems. She presented with cyclical vomiting for 3 years duration. There can be multiple causes for vomiting. Vomiting usually disturbs the acid base and electrolyte balance of the body. Our case illustrates that uncommon aetiology could be the reason for common clinical presentations and investigation abnormalities. Identifying the correct aetiology is lifesaving.

A few case reports have shown that patients with adrenal insufficiency present with intractable nausea and vomiting [3]. However, this is an atypical rare clinical presentation of a nonfunctioning pituitary macroadenoma in which the patient presented with cyclical vomiting. This case also highlights that, despite having several psychosocial issues, an organic pathology should always be sought before attributing the unexplained symptoms to a psychological illness. Hence, we decided to report this case in order to share our clinical experience.

## 2. Case History

A 23-year-old Sri Lankan female university student presented to the medical casualty ward with several episodes of vomiting for two days duration. She had vomited 5-6 episodes per day, which was associated with burning-type chest pain and regurgitation. Vomiting was a nonprojectile, nonbilious, and nonfecal in nature. There was no hematemesis. Vomiting was preceded by nausea and she had tried to induce vomiting by digital stimulation of the throat. There was no history of fever, abdominal pain, dysuria, abdominal distention, or altered bowel habits. However, she complained of reduced appetite and generalized body weakness for 3 years duration and postural dizziness for the last 2 days. She denied associated headache, photophobia, visual disturbances, and weight loss. Further questioning of her past history revealed frequent hospital visits due to similar type of recurrent episodes of vomiting over the last 3 years. These episodes always followed the same pattern with sudden onset of vomiting preceded by nausea lasting for 4-5 days. There were 4-5 episodes of vomiting per day. In each episode of vomiting, she was managed as having gastroesophageal reflux disease with proton pump inhibitors and prokinetics as an outpatient without being investigated thoroughly. In between these episodes, she was perfectly normal and engaged in her usual day-to-day activities. The first episode occurred 3 years earlier. She attributed its onset to the breakup of her relationship. Initially, the vomiting frequency was less, around 1-2 episodes in every 6 months. However, the frequency had increased recently. Last hospital visit due to recurrent vomiting took place 3 months earlier. However, we were unable to find her past medical records of her previous hospital visits.

On further inquiring, she denied excessive exercise and use of appetite suppressants and diuretics which are common with eating disorders. She did not have ideas of body-image distortion. She denied intake of large volume of water. There was no history of polyuria, polydipsia, or nocturia.

She had attained menarche at the age of 14 years and developed secondary amenorrhea since the age of 20 years. She denied galactorrhea. She was on anxiolytics for acute stress reaction following her relationship breakup 5 years earlier. Anxiolytics was taken only for one-month duration.

Onset of recurrent episodes of cyclical vomiting and amenorrhea all had occurred in 2016, soon after she started her university life.

She disclosed that her mother had died from an astrocytoma 7 years ago. She lived with her siblings, father, and stepmother.

On examination, her Glasgow coma scale was 15/15. She had a low BMI, 16 kg/m^2^. Her pulse rate was 78 beats/minute and blood pressure was 95/60 mmHg. There were no signs suggestive of an eating disorder such as dental caries, erosions over knuckles of hands, parotid enlargement, or brittle hair. Rest of her system examination was normal.

### 2.1. Investigations

She had normal capillary blood glucose level on admission (80 mg/dl). Her white cell count, haemoglobin, platelet, liver transaminases, alkaline phosphate, serum albumin, serum amylase level, and total bilirubin level were normal. Her chest X-ray, electrocardiogram, and urine analysis were normal. Her total serum beta HCG level was normal. C-reactive protein was 5 mg/l and ESR was 10 mm.

However, she had markedly deranged serum electrolyte levels. Her serum sodium was 110 mmol/L (136–145 mmol/L). Serum potassium and chloride were 3.2 mmol/L (3.5–5.3 mmol/L) and 74 mmol/L (97–111 mmol/L), respectively. Repeated serum electrolyte showed similar low values. Arterial blood gas analysis showed respiratory alkalosis (PH, 7.481; PCO_2_, 24 mmHg; PO_2_, 125 mmHg; and bicarbonate level, 17 mmol/L) probably due to stress-induced hyperventilation. Her blood urea was normal (14 mg/dl). Serum creatinine was 44.9 *µ*mol/L. Serum osmolality was found to be 223 mOsm/kg and urine osmolality was 266 mOsm/kg. Her average urine output was 1050/day. Urinary electrolyte studies showed low urinary potassium and sodium levels, 5.3 mmol/L and 14 mmol/L, respectively. Urine chloride level was 24 mmol/L.

Observed electrolyte abnormalities were evaluated thoroughly. Her random serum cortisol level (105 nmol/l) which was taken on admission day at 1 a.m. was low. Vomiting is an acute stress to the body. It usually causes high cortisol level in the body. This result contradicted it. She also had low thyroid stimulating hormone (TSH) of 0.209 *µ*IU/ml (0.55–4.78 *µ*IU/ml), low free triiodothyronine (*T*_3_) of 1.32 pg/ml (2.30–4.20 pg/ml), and low normal free tetraiodothyronine (*T*_4_) level of 0.98 ng/dl (0.93–1.7 ng/dl).

With the background history of generalized body weakness, reduced appetite, and secondary amenorrhea, she was investigated further. Her both follicular stimulating hormone (FSH, 1.4 mIU/ml) and luteinizing hormone (LH, 0.4 mIU/ml) were found to be low. Ultrasound abdomen scan showed a hypoplastic uterus. There were no other abnormalities in the ultrasound scan of abdomen. Her prolactin level was found to be mildly elevated. Serum prolactin level was 81 ng/ml (nonpregnant range, 2–29.2 ng/ml) and with 5 : 1 dilution it was 90.8 ng/ml. Summary of blood and urine investigation findings is shown in Table 1.

She had biochemical evidence of hypoadrenalism, possible central hypothyroidism, hypogonadotropic hypogonadism, and mild hyperprolactinemia.

**Figure 1:**
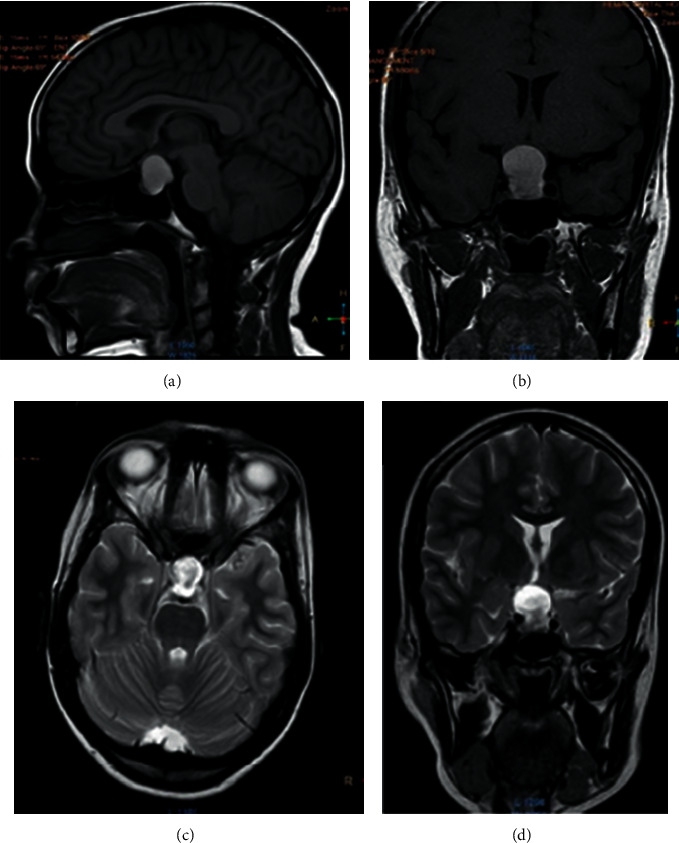
MRI of this patient showing lobular tumor occupying sellar and suprasellar region measuring 2.4  cm × 2.0 cm × 2.0 cm. Caudal part of the lesion showed intermediate signal intensity in T2-weighted images. Superior mass effect is seen on the optic chiasma, optic tract, and AI segments of bilateral anterior cerebral arteries. Mass is bulged into the sphenoid sinuses. ((a, b)) T1-weighted, gadolinium-enhanced sagittal and coronal views. ((c, d)) T2-weighted axial and coronal views.

To assess her hypothalamic-pituitary axis further, magnetic resonance imaging (MRI) of pituitary was done. MRI scan showed 2.4 cm × 2 cm × 2 cm sized lobular mass occupying sellar and suprasellar area with mass effect on optic chiasma, optic tracts, and AI segment of bilateral anterior cerebral arteries. There was enlargement of sellar by the lesion caudally and the mass has bulged into the sphenoid sinus. There were scattered hemorrhages on the mass (Figure 1). Humphrey field analyzer threshold plot demonstrated bitemporal hemianopia. The diagnosis of a pituitary nonfunctioning macroadenoma with mass effect causing hypoadrenalism, central hypothyroidism, hypogonadotropic hypogonadism, and hyperprolactinemia due to stalk effect was made.

### 2.2. Differential Diagnosis

The differential diagnosis made on admission is illustrated in Table 2.

### 2.3. Management, Outcome, and Follow-Up

On admission, she was managed with antiemetics. Volume loss due to vomiting was replaced with equal volumes of oral rehydration fluid. Since the patient tolerated very low level of serum sodium, she was managed as having chronic hyponatremia worsened further by recurrent vomiting and reduced intake of salt. Therefore, aggressive management was not done to correct hyponatremia. Intravenous hydrocortisone 100 mg bolus dose was given suspecting hypoadrenalism and continued intravenous hydrocortisone 50 mg every 6 hours. Low serum potassium and chloride levels were thought to be due to vomiting, which got corrected after oral hydration. She had respiratory alkalosis due to stress-induced hyperventilation.

After making a diagnosis of a nonfunctioning pituitary macroadenoma with hormonal deficiency and mass effect, the patient was started on levothyroxine 50 *µ*g daily while continuing intravenous hydrocortisone 50 mg every 6 hours. Four days after hormonal replacement, her serum sodium level became stable around 132 mmol/L. Her vomiting improved. There was marked improvement of her generalized body weakness and appetite. The intravenously administered hydrocortisone was then converted to oral hydrocortisone and after 2 weeks of treatment there was further improvement. One month later, her serum-free T4 was 0.81 ng/dl (0.93–1.7 ng/dl).

Transsphenoidal resection of the pituitary tumor was performed one month after the diagnosis. Histology revealed a pituitary adenoma. The first twenty-four hours of postoperative period were covered with intravenous hydrocortisone 100 mg every 6 hours and the same dose of levothyroxine. The postoperative period was complicated with transient diabetes insipidus, which was managed with intravenous vasopressin followed by desmopressin nasal spray.

She was discharged with 50 *µ*g of levothyroxine and orally administered total 20 mg daily dose of hydrocortisone. Currently, her general condition is stable with serum sodium 136 mmol/L and free *T*_4_ 0,96 ng/dl. She was free of symptoms suggestive of hypoadrenalism. MRI pituitary performed five months after the surgery showed no residual tumor. Interestingly, she recovered from her episodes of recurrent vomiting following hormone replacement and pituitary surgery.

## 3. Discussion

Pituitary tumors account for 15.5% of all central nervous system (CNS) neoplasms [1, 5]. In young adults (20–34 years), 30% of CNS tumors are due to pituitary adenomas [1, 5]. Nonfunctioning pituitary adenomas account for a mean of 33% of all pituitary adenomas, which are lower than those of prolactinomas, which account for 47% of all pituitary adenomas [1, 6]. Nonfunctioning pituitary adenomas commonly appear as pituitary macroadenomas [1]. Their symptoms and signs are usually due to anterior pituitary hormone deficiency and mass effect on adjacent structure, especially on optic chiasma, occurring in 60%–80% of cases [1, 2].

Most of patients with nonfunctioning pituitary adenomas have one or more anterior pituitary hormone deficiencies [1, 7, 8]. More than 20% of patients with nonfunctioning pituitary adenoma have two or more hormone deficiencies [1, 7]. 40%–75% of patients with nonfunctioning pituitary adenoma can have hypogonadotropic hypogonadism, and 20%–40% of cases develop central hypothyroidism and hypocortisolism [1, 7]. There can be mild hyperprolactinemia (<100 mg/ml) due to the compression of pituitary stalk by the tumor interrupting the descending dopaminergic effects [1].

8%–10% of pituitary adenomas can present as pituitary apoplexy [7]. Pituitary apoplexy is due to infarction or hemorrhage of the pituitary tumor that is characterized by sudden onset headache, vomiting, altered level of consciousness, hemodynamic instability, and visual abnormalities [1, 7].

In this case, the patient presented with atypical symptoms of nonfunctioning pituitary macroadenoma, though she had biochemical evidence supportive of a pituitary macroadenoma. She presented with recurrent vomiting in the background of cyclical episodes of vomiting with intermittent vomiting-free periods. She complained of generalized body weakness and reduced appetite, which might be nonspecific symptoms associated with hypoadrenalism. She had secondary amenorrhea suggestive of involvement of the hypothalamic-pituitary axis. Our patient's presentation is different from the typical presentation seen in pituitary apoplexy, although there were scattered hemorrhages in the lesion in MRI study.

Our patient had various psychosocial issues such as loss of the mother at young age, relationship breakup, and stresses from university life. The patient attributed onset of all her symptoms following her relationship breakup which occurred soon after she entered the university. Having several psychosocial problems could mislead the doctors from the correct diagnosis pathway by attributing all nonspecific, unexplained symptoms to a psychological illness. Although could have been having coexistent psychological issues, before making a psychological diagnosis, it is mandatory to exclude organic causes. This case highlights the importance of excluding organic causes before arriving at a psychological diagnosis.

Severe hyponatremia was another rare presentation of a nonfunctioning pituitary macroadenoma, which was observed in this case. With the history of recurrent vomiting, it is likely that one can mistakenly take vomiting as the sole cause for the observed electrolyte imbalance. This case highlights the importance of evaluating an electrolyte imbalance thoroughly because there can be multiple underlying aetiologies.

Only a few case reports have shown severe hyponatremia as a presenting symptom of a pituitary macroadenoma as in this case [4]. Therefore, high index of suspicion is needed when evaluating chronic or persistent hyponatremia [4].

Hyponatremia is defined as a serum sodium level less than 135 mmol/L. Severe hyponatremia is when serum sodium is below 125 mmol/L, which is associated with increased morbidity and mortality [4]. An approach to the evaluation of hyponatremia involves history taking, examination of each system of body, taking history of diuretics or any other drug use, serum, and urine biochemical tests. It is necessary to evaluate volume status, osmolality levels of the body, and urinary sodium level.

Hyponatremia due to hypopituitarism is usually due to secondary hypoadrenalism rather than central hypothyroidism [9]. Hyponatremia due to secondary hypoadrenalism is caused by impaired electrolyte-free water excretion in the absence of normal cortisol activity in the kidney [9]. Also, there is increased secretion of arginine vasopressin, a secondary ACTH secretagogue, which results in urinary concentration and can further impair hyponatremia [9].

Alteration in renal perfusion and reduced glomerular filtration are seen in hypothyroidism. Hyponatremia due to hypothyroidism is due to inability to excrete free water. There is effect of arginine vasopressin on dilution of urine. Reduced glomerular filtration rate in hypothyroidism diminishes free water excretion by reducing water delivery to the tubules, which causes hyponatremia [9].

Unlike in primary hypoadrenalism, in secondary hypoadrenalism, since there is no mineralocorticoid deficiency, it is unlikely that patients develop hyperkalemia.

Protracted vomiting without replacement of fluid ideally should cause volume depletion and hypernatremia [9]. However, if the patient takes fluid and food low in sodium content and in conjunction with a baroreceptor mediated stimulus to arginine vasopressin secretion which is an antidiuretic hormone, this can result in hyponatremia with vomiting and diarrhea [9]. History and examination are needed in evaluating hyponatremia due to vomiting and diarrhea. Symptoms and signs of volume depletion can be seen [9]. The urine sodium will be low with volume depletion but, with ongoing vomiting, it can be high due to urinary loss of bicarbonate, which causes excretion of an accompanying cation [9]. However, low urinary chloride level is a reliable indicator of volume depletion with vomiting [9].

It is clinician's duty to find correct aetiology of hyponatremia. Our patient had severe hyponatremia with minimal hyponatremic symptoms due to its chronic nature. However, having low serum potassium and serum chloride level with low urinary sodium and potassium levels highlighted that her hyponatremia was worsened by ongoing vomiting episodes and by reduced intake of salts due to reduced appetite. She denied polydipsia and polyuria, and her urine output was normal. She had low plasma osmolality with mildly elevated urine osmolality. Her blood urea level was normal. This led to the evaluation to find aetiology for hidden euvolemic chronic hyponatremia. This led to the diagnosis of underlying pituitary macroadenoma. Observed low serum potassium and chloride were attributed due to vomiting and reduced intake of salt.

This is a very atypical presentation of a patient with a nonfunctioning pituitary adenoma who presented with cyclical vomiting with severe hyponatremia. After correcting her hormone deficiencies and undergoing pituitary surgery, she did not develop any episode of recurrent vomiting.

## 4. Conclusion

Although nonfunctioning pituitary macroadenomas generally present with symptoms and signs of anterior pituitary hormone deficiencies and mass effect on adjacent structures, they can also have unusual presentations. Cyclical vomiting and severe hyponatremia are atypical presentations of nonfunctioning pituitary macroadenomas. Overlapping multiple aetiologies can be found in a patient with recurrent vomiting and hyponatremia. Identification of the correct aetiology is lifesaving. Therefore, high level of suspicion is needed when evaluating recurrent vomiting and hyponatremia.

## Figures and Tables

**Table 1:** Summary of blood and urine investigation findings.

Name of the investigation	Value	Name of the investigation	Value
White cell count	6000 cells/mm3	Serum creatinine	44.9 *µ*mol/L
Neutrophil	81%	PH	7.481
Lymphocyte	16%	PCO_2_	24 mmHg
Haemoglobin	12.3 g/dl	PO_2_	125 mmHg
Platelet	244 000/mm3	Bicarbonate level	17 mmol/L
		Blood urea	14 mg/dl
Serum sodium	110 mmol/L (136–145 mmol/L)		
Serum potassium	3.2 mmol/L (3.5–5.3 mmol/L)	Her random serum cortisol level	105 nmol/l
Serum chloride	74 mmol/L (97–111 mmol/L)	TSH	0.209 *µ*IU/ml (0.55–4.78 *µ*IU/ml)
Plasma osmolality	223 mOsm/kg	T3 level	1.32 pg/ml (2.30–4.20 pg/ml)
Urine osmolality	266 mOsm/kg	T4 level	0.98 ng/dl (0.93–1.7 ng/dl).
Urine sodium	14 mmol/L	LH level	0.4 mIU/ml
Urine potassium	5.3 mmol/L	FSH level	1.4 mIU/ml
Urine chloride	24 mmol/L	Serum prolactin level	81 ng/ml (nonpregnant range, 2.–29.2 ng/ml)
		Serum prolactin level (5 : 1 dilution)	90.8 ng/ml.
Alanine transaminase	23.4 U/l (7–35 U/l)	Total serum beta HCG	<2 mIU/ml (<10)
Aspartate transaminase	20.3 U/l (0–31 U/l)		
Alkaline phosphatase	68.2 U/l (30–120 U/l)	Urine full report	Normal
Total protein	6.5 g/dl (6–8 g/dl)		
Serum albumin	4.1 g/dl (3.7-5 g/dl)		
Adjusted calcium	8.7 mg/dl (8.6–10 mg/dl)		
Serum magnesium	0.9 mmol/L (0.7–1 mmol/l)		
Serum inorganic phosphorous	3 mg/dl (2.7–4.5 mg/dl)		
Serum amylase	32 U/l (30–110 U/l)		
C-reactive protein	5 mg/l		
ESR	10 mm		

**Table 2:** Differential diagnosis.

(1) Recurrent vomiting due to gastroesophageal reflux disease or any other abdominal pathology in the abdomen
(2) Chronic infection, e.g., pyelonephritis
(3) Eating disorder, e.g., anorexia nervosa
(4) Syndrome of inappropriate antidiuretic hormone secretion (SIADH)
(5) Adrenal insufficiency
(6) Cyclical vomiting syndrome
(7) Psychogenic vomiting

## Data Availability

The clinical data are available in the clinical records of the patient, which are stored in the record room of Sri Jayewardenepura General Hospital, Nugegoda, Colombo, Sri Lanka.
